# Author Correction: Diagnosis of fusion genes using targeted RNA sequencing

**DOI:** 10.1038/s41467-020-15697-9

**Published:** 2020-04-08

**Authors:** Erin E. Heyer, Ira W. Deveson, Danson Wooi, Christina I. Selinger, Ruth J. Lyons, Vanessa M. Hayes, Sandra A. O’Toole, Mandy L. Ballinger, Devinder Gill, David M. Thomas, Tim R. Mercer, James Blackburn

**Affiliations:** 1 Genomics and Epigenetics Division, Garvan Institute of Medical Research, Sydney, 2010 NSW Australia; 2 St. Vincent’s Clinical School, UNSW Australia, Sydney, 2031 NSW Australia; 3 Tissue Pathology and Diagnostic Oncology, Royal Prince Alfred Hospital, Sydney, 2050 NSW Australia; 4 Faculty of Health Sciences, University of Limpopo, Turfloop Campus, Mankweng, 0727 South Africa; 5 School of Health Systems and Public Health, University of Pretoria, Pretoria, 0002 South Africa; 6 Central Clinical School, University of Sydney, Sydney, 2006 NSW Australia; 7 The Kinghorn Cancer Centre and Cancer Division, Garvan Institute of Medical Research, Sydney, 2010 NSW Australia; 8 Australian Clinical Labs, Sydney, 2010 NSW Australia; 9 Department of Haematology, Princess Alexandra Hospital, Brisbane, 4102 QLD Australia; 10 Altius Institute for Biomedical Sciences, Seattle, 98121 WA USA

**Keywords:** RNA sequencing, Cancer, Cancer genomics, Molecular medicine

Correction to: *Nature Communications* 10.1038/s41467-019-09374-9, published online 27 March 2019.

The original version of the Supplementary Information associated with this Article did not include Supplementary Figure 13. The HTML has been updated to include a corrected version of the Supplementary Information.

