# Supplementary material for: Diagnosis of fusion genes using targeted RNA sequencing
Source: Nat Commun. 2019 Mar 27;10:1388. doi: 10.1038/s41467-019-09374-9 (PMC6437215; doi:10.1038/s41467-019-09374-9)
Supplement: Supplementary file 1 — Supplementary Information [file 41467_2019_9374_MOESM1_ESM.pdf]

SUPPLEMENTARY MATERIALS

Diagnosis of fusion genes using targeted RNA sequencing

Heyer *et al.*

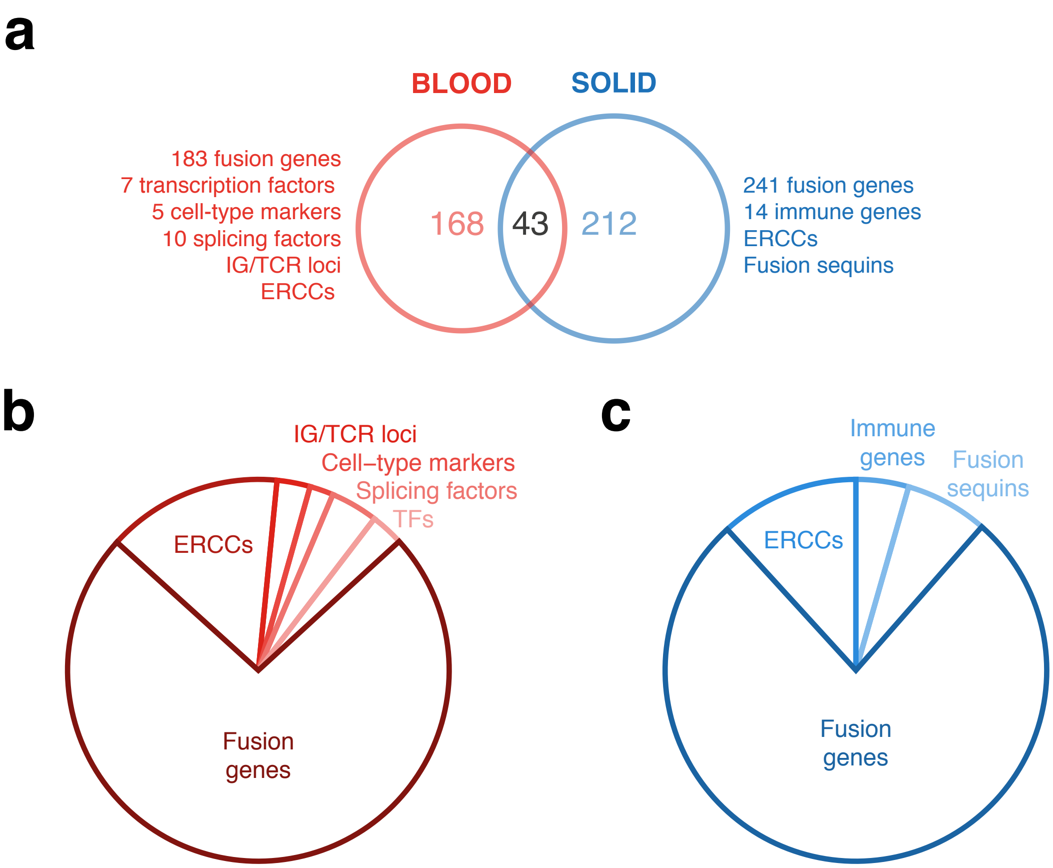

Supplementary Figure 1. Overview of targeted RNAseq panel designs. **a)** A Venn diagram summarizing the relative sizes and overlap of the blood and solid targeted sequencing panels. **b)** Distribution of target genes on the blood panel. **c)** Distribution of target genes on the solid panel.

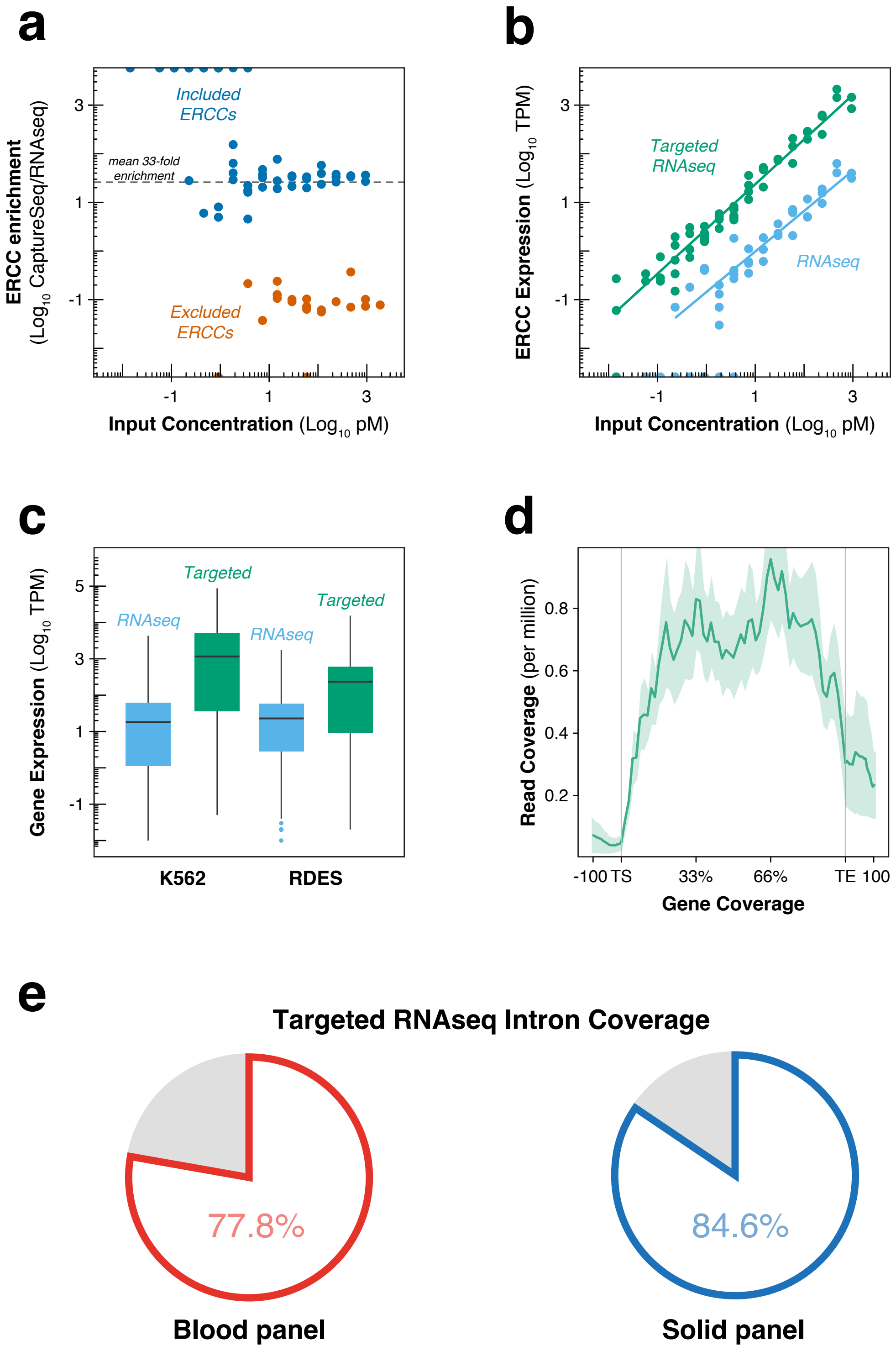

Supplementary Figure 2. Targeted RNAseq panel validation. **a)** Scatterplot of targeted RNAseq enrichment for ERCCs included (blue) or excluded (orange) on the solid panel. **b)** Expression levels of targeted ERCCs in conventional RNAseq compared to targeted RNAseq with the solid panel. **c)** Boxplot comparing gene expression levels in conventional RNAseq versus targeted RNAseq for both blood (K562) and solid (RDES) panels. The lower and upper hinges correspond to the 25th and 75th percentiles, respectively; middle lines correspond to the median; whiskers extend from hinges to the smallest or largest value no further than 1.5*IQR(inter-quartile range). **d)** Metagene plot of RDES targeted RNAseq read coverage across all genes on the solid panel. TS = Transcription Start site; TE = Transcription End site. **e)** Percentage of on-panel annotated introns covered by splice-junction reads on the blood (K562) or solid (RDES) panels.

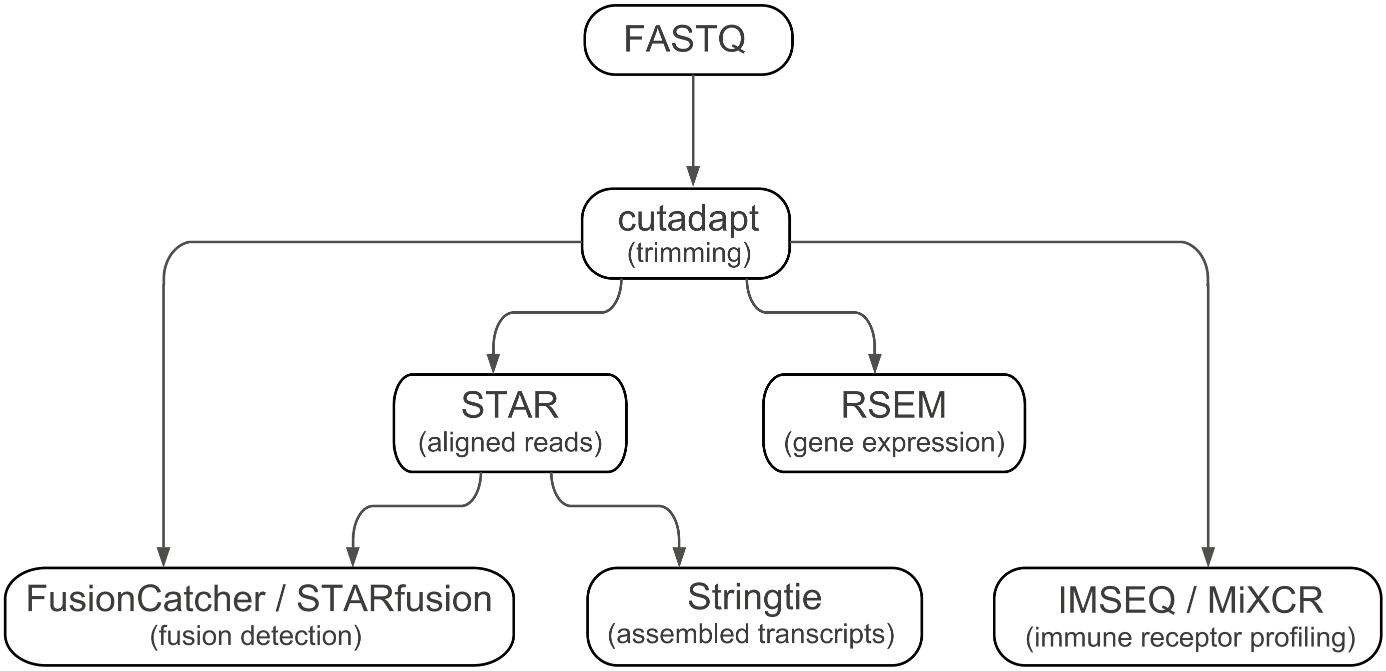

Supplementary Figure 3. Schematic of bioinformatic analytical pipeline.

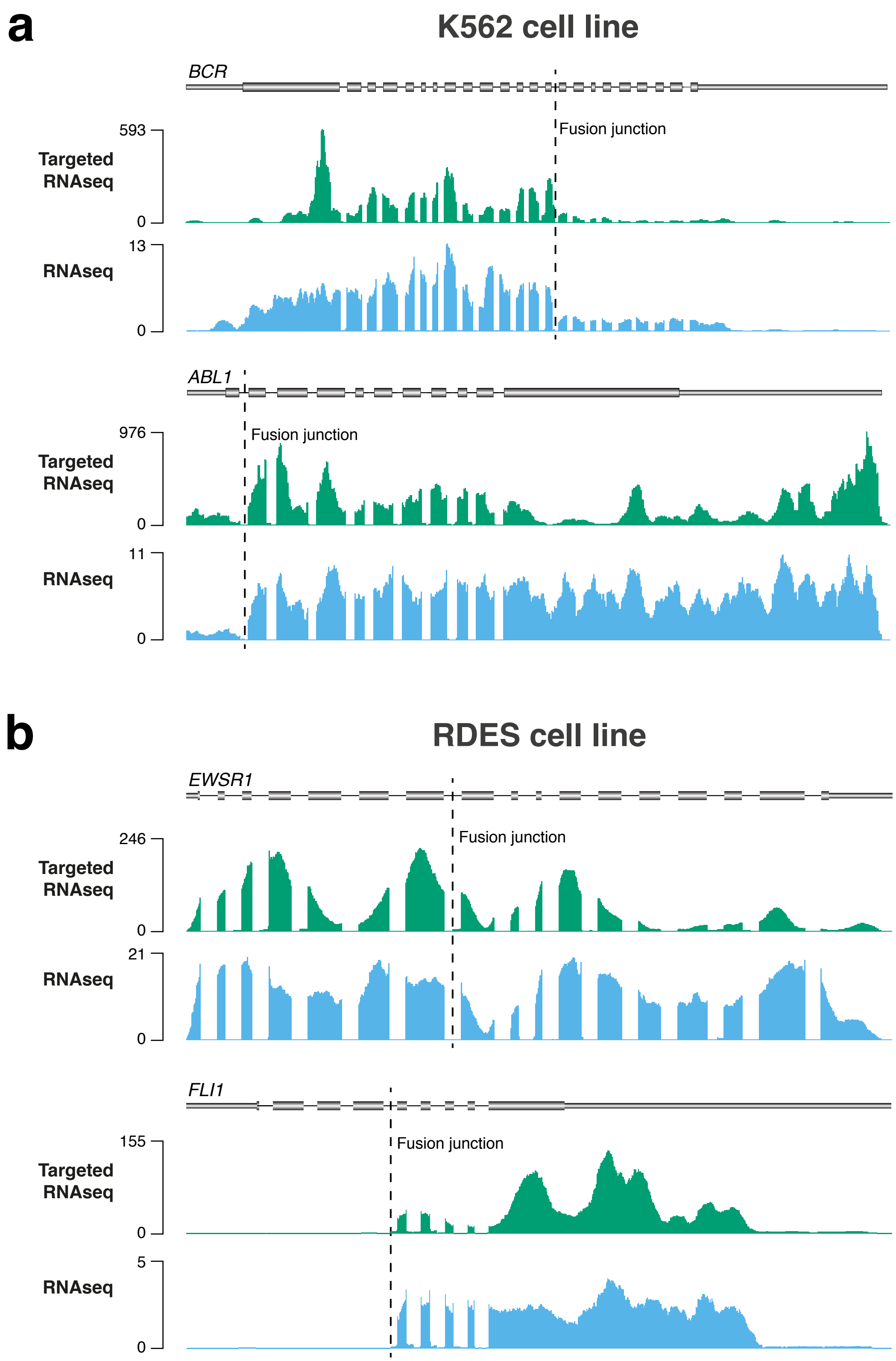

Supplementary Figure 4. Read coverage across fusion genes in cell lines. **a)** Genome browser screenshot showing enhanced targeted RNAseq read coverage compared to canonical RNAseq for *BCR* and *ABL1* genes. Dotted line marks location of fusion junction. **b)** Genome browser screenshot showing enhanced targeted RNAseq read coverage compared to canonical RNAseq for *EWSR1* and *FLI1* genes. Dotted line marks location of fusion junction.

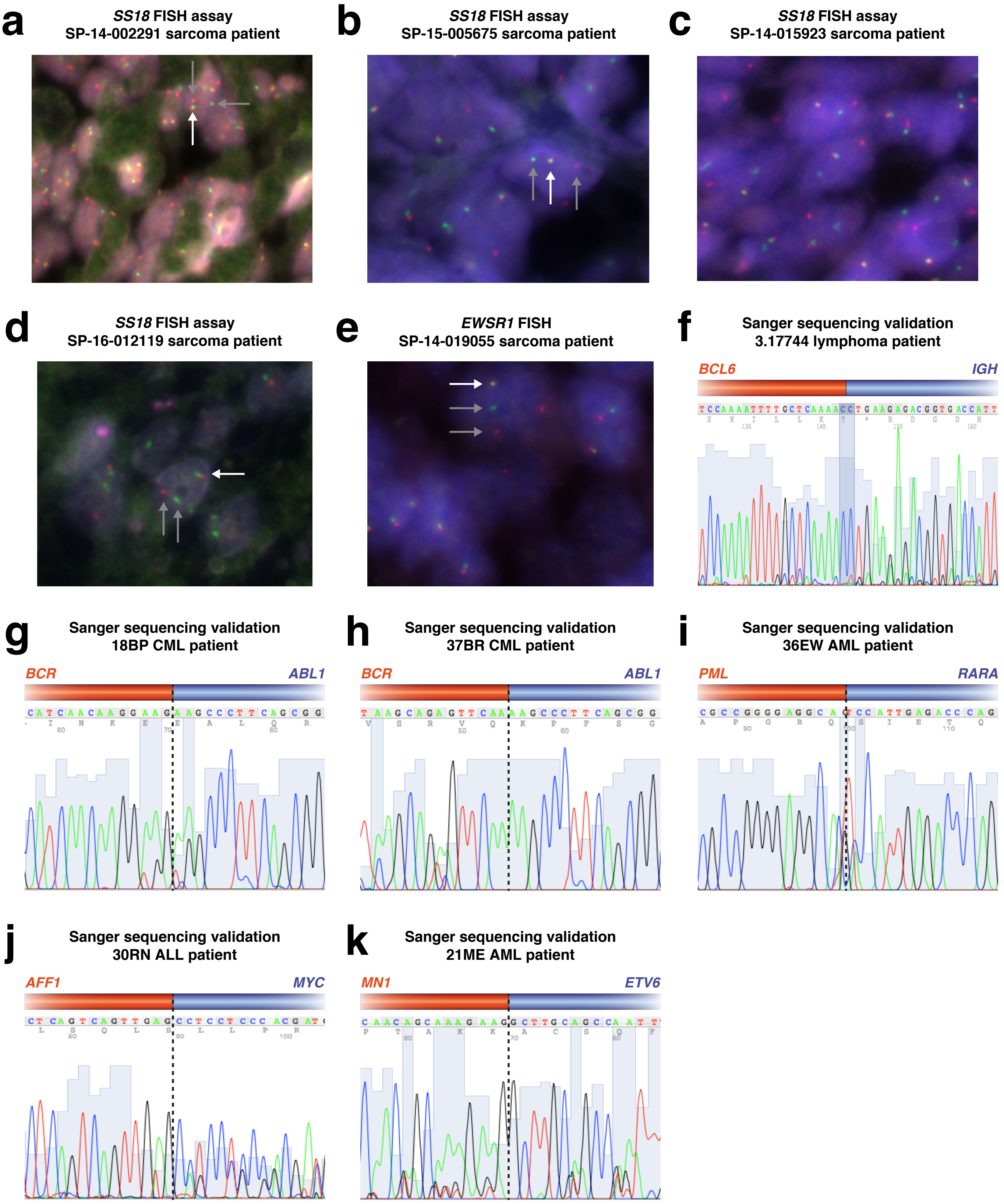

Supplementary Figure 5. Clinical fusion gene identification and validation. **a-e)** FISH fusion detection using breakapart probes for *SS18* in samples SP-14-002291 **(a)**, SP-15-005675 **(b)**, SP-14-015923 **(c)** and SP-16-012119 **(d)** and *EWSR1* in sample SP-14-019055. *EWSR1* rearrangement detected in 30% of cells **(e)**. Positive signal demonstrated by 1 fused probe set (white arrow) and 1 isolated red and 1 isolated green dot (grey arrows). **f-k)** Sanger sequencing validation of *IGH-BCL6* fusion gene in lymphoma patient 3.17744 **(f)**, *BCR-ABL1* fusion gene in CML patient 18BP **(g)**, *BCR-ABL1* fusion gene in CML patient 37BR **(h)**, *PML-RARA* fusion gene in AML patient 36EW **(i)**, *AFF1-MYC* fusion gene in ALL patient 30RN **(j)**, *MN1-ETV6* fusion gene in AML patient 21ME **(k)**.

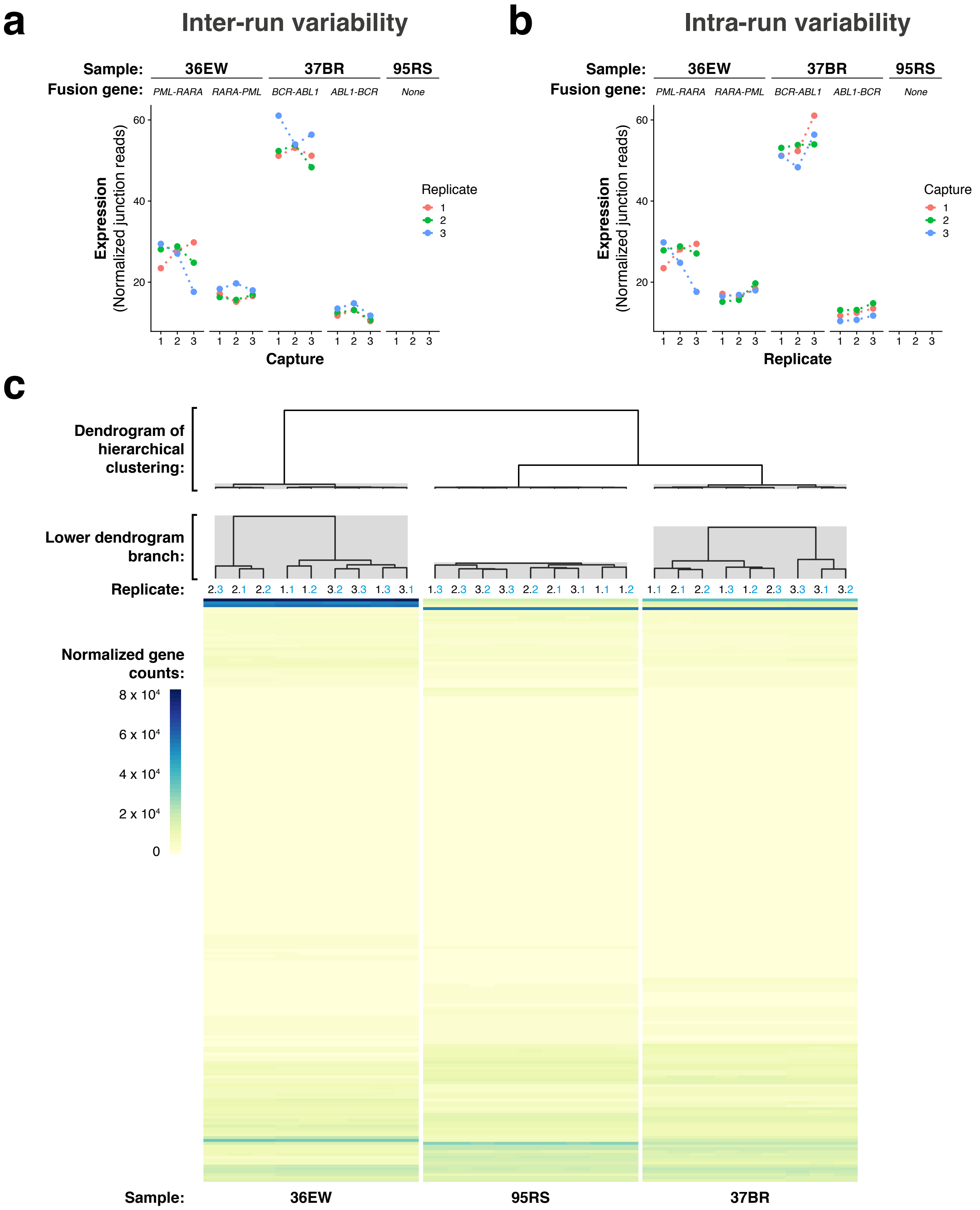

Supplementary Figure 6. Measuring reproducibility of targeted RNAseq assay. **a-b)** Scatterplots of fusion junction reads comparing either **(a)** replicates between individual capture events to visualize inter-run variability or **(b)** replicates within each capture event to visualize intra-run variability. **c)** Hierarchical clustering of gene expression for all genes captured on the blood panel. Top panel: dendrogram representing clustering between the samples. Middle panel: zoom of the lower dendrogram branch (indicated by grey boxes). Numbers indicate replicate and capture number per sample. Bottom panel: gene expression heatmap generated with read counts per gene normalized to library size; each row represents one gene.

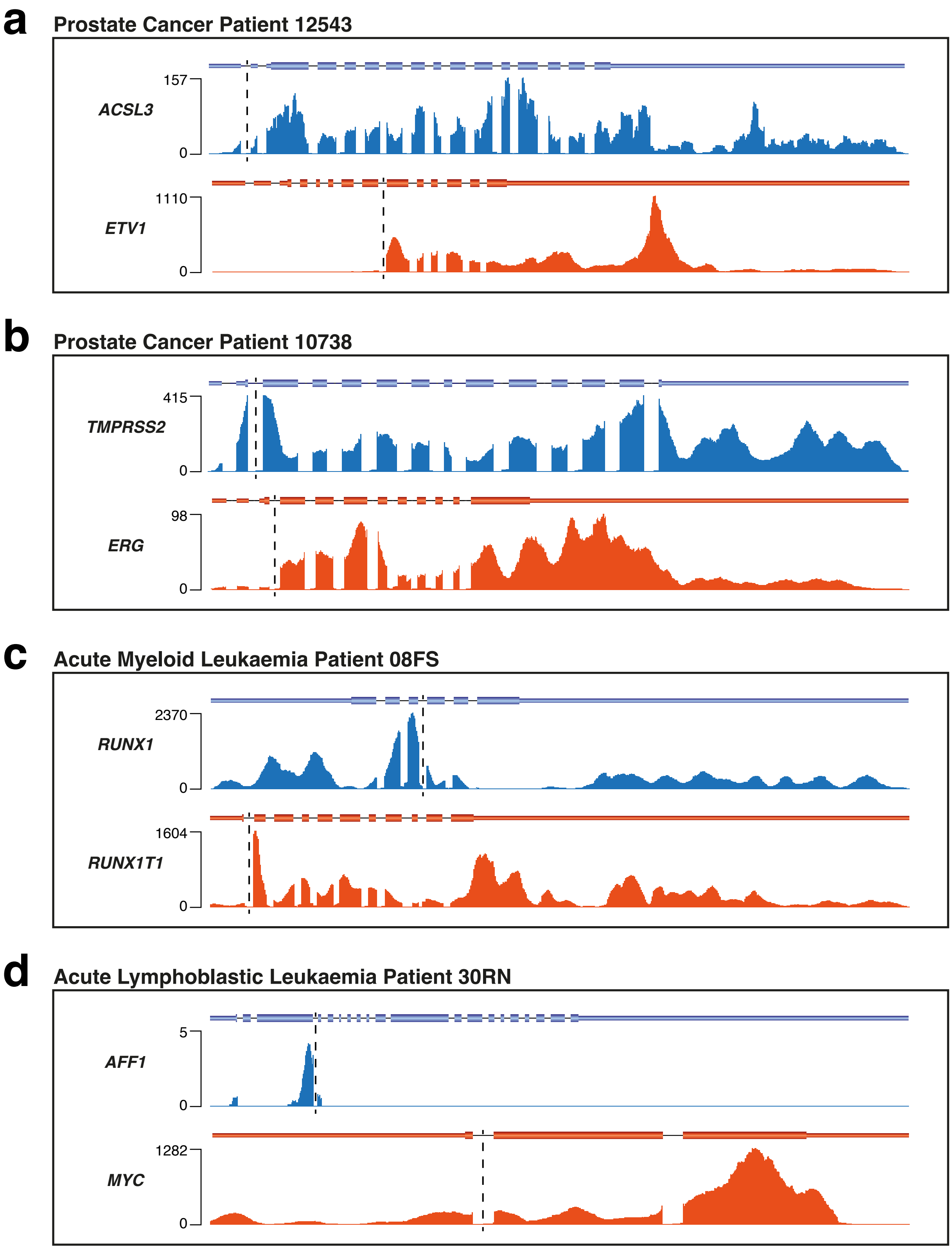

Supplementary Figure 7. Examples of targeted RNAseq read coverage across fusion genes in clinical cohort samples. **a)** Read coverage across *ACSL3* and *ETV1* genes in prostate cancer patient sample 12543. Dotted line marks fusion junction of *ACSL3-ETV1* fusion gene. **b)** Read coverage across *TMPRSS2* and *ERG* genes in prostate cancer patient sample 10738. Dotted line marks fusion junction of *TMPRSS2-ERG* fusion gene. **c)** Read coverage across *RUNX1* and *RUNX1T1* genes in AML patient sample 08FS. Dotted line marks fusion junction of *RUNX1-RUNX1T1* fusion gene. **d)** Read coverage across *AFF1* and *MYC* genes in ALL patient 30RN. Dotted line indicates location of *AFF1-MYC* fusion junction.

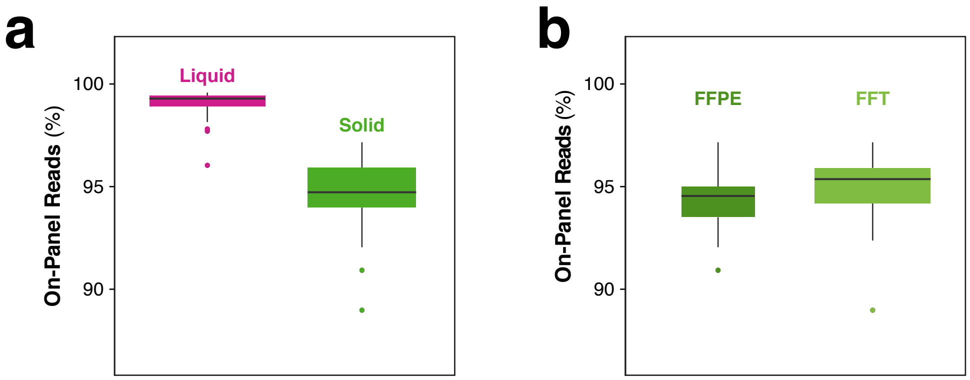

Supplementary Figure 8. Effects of sample source on alignment mapping. **a)** Boxplot comparing on-panel mapping percentages for sample types (Liquid = bone marrow aspirate and peripheral blood; Solid = FFPE and fresh-frozen tissue (FFT)). p = 5.8 x 10^{-16}. **b)** Boxplot comparing on-panel mapping percentages versus tissue type. p = 0.50. p-values calculated using Wilcoxon rank sum test. For both plots, the lower and upper hinges correspond to the 25th and 75th percentiles, respectively; middle lines correspond to the median; whiskers extend from hinges to the smallest or largest value no further than 1.5*IQR(inter-quartile range).

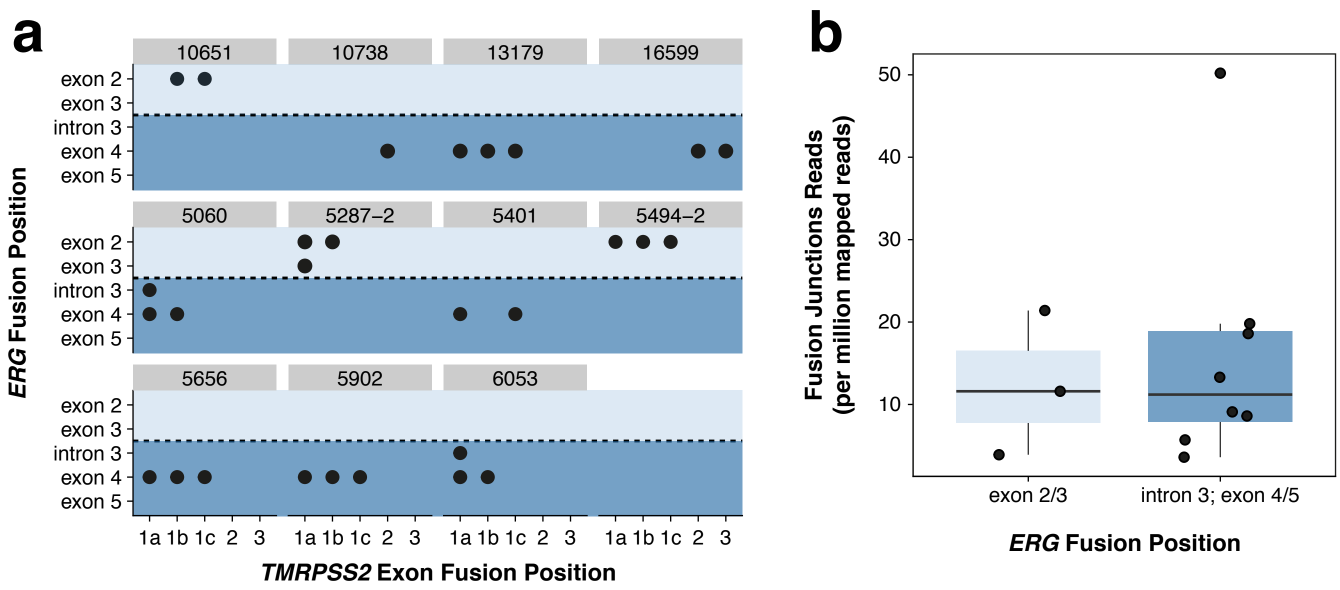

Supplementary Figure 9. Effect of fusion location on transcript expression. **a)** Fusion junction position between *TMPRSS2* and *ERG* shown for each *TMPRSS2-ERG* positive prostate cancer patient. Light and dark blue background distinguishes proximal and distal *ERG* fusion junction positions. **b)** Boxplots with datapoints overlaid demonstrating no variation in fusion transcript expression correlating to proximal versus distal *ERG* fusion junction position. The lower and upper hinges correspond to the 25th and 75th percentiles, respectively; middle lines correspond to the median; whiskers extend from hinges to the smallest or largest value no further than 1.5*IQR(inter-quartile range).

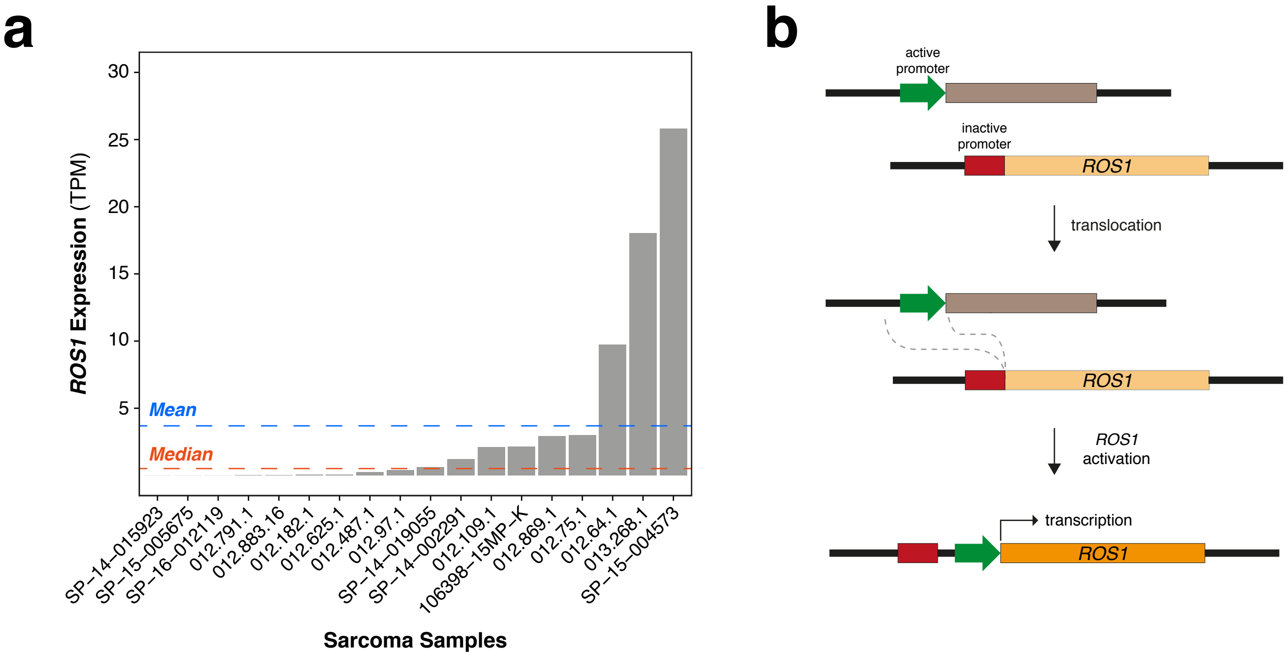

Supplementary Figure 10. *ROS1* expression and rearrangement. **a)** *ROS1* gene expression levels throughout all sarcoma samples. Blue dotted line indicates mean; orange dotted line indicates median. **b)** Schematic showing how a chromosomal translocation could result in a *ROS1* promoter fusion.

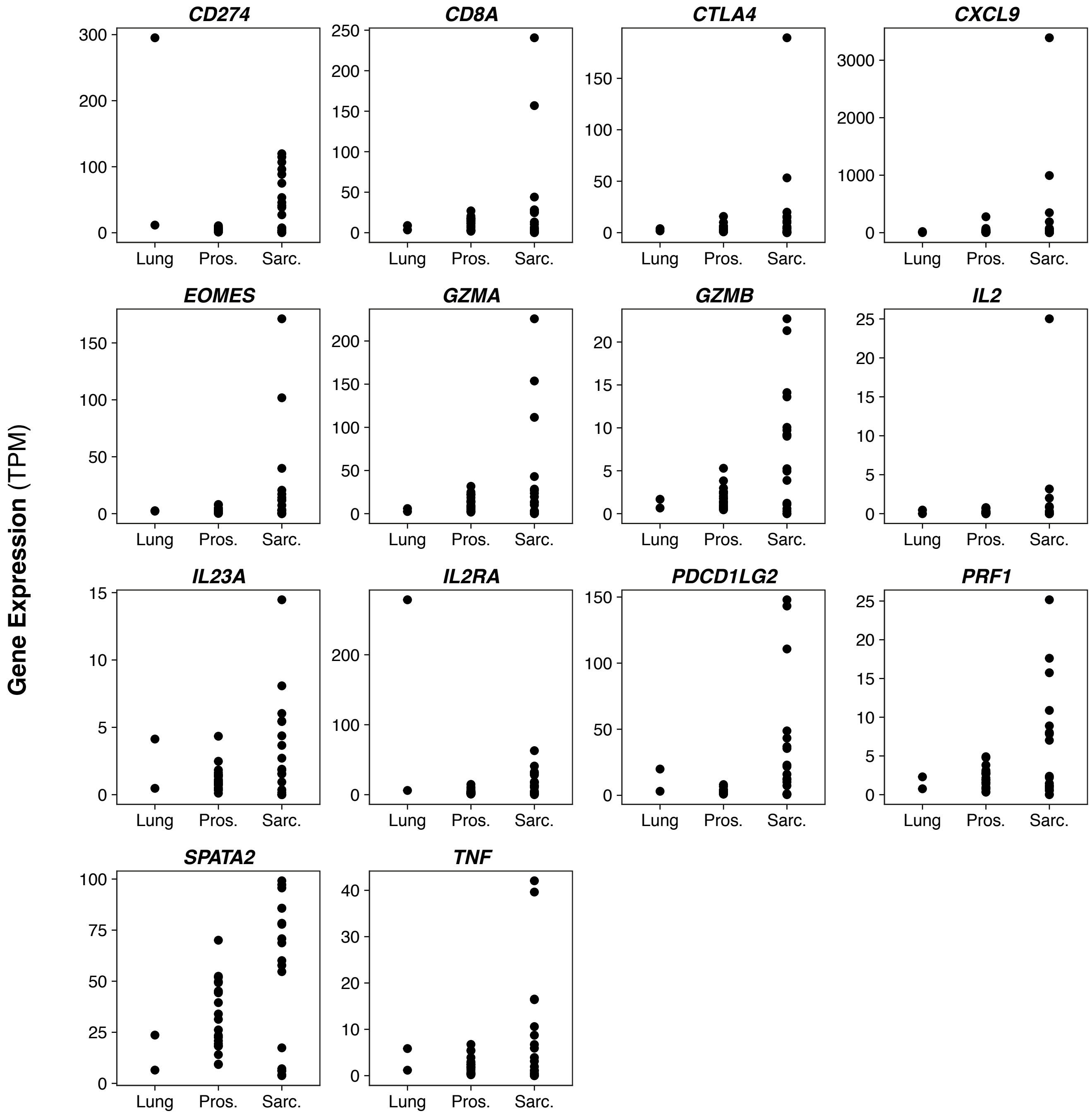

Supplementary Figure 11. Marker gene expression in clinical cohort samples processed on the solid panel. Lung = lung cancer patient samples; Pros. = prostate cancer patient samples; Sarc. = sarcoma patient samples.

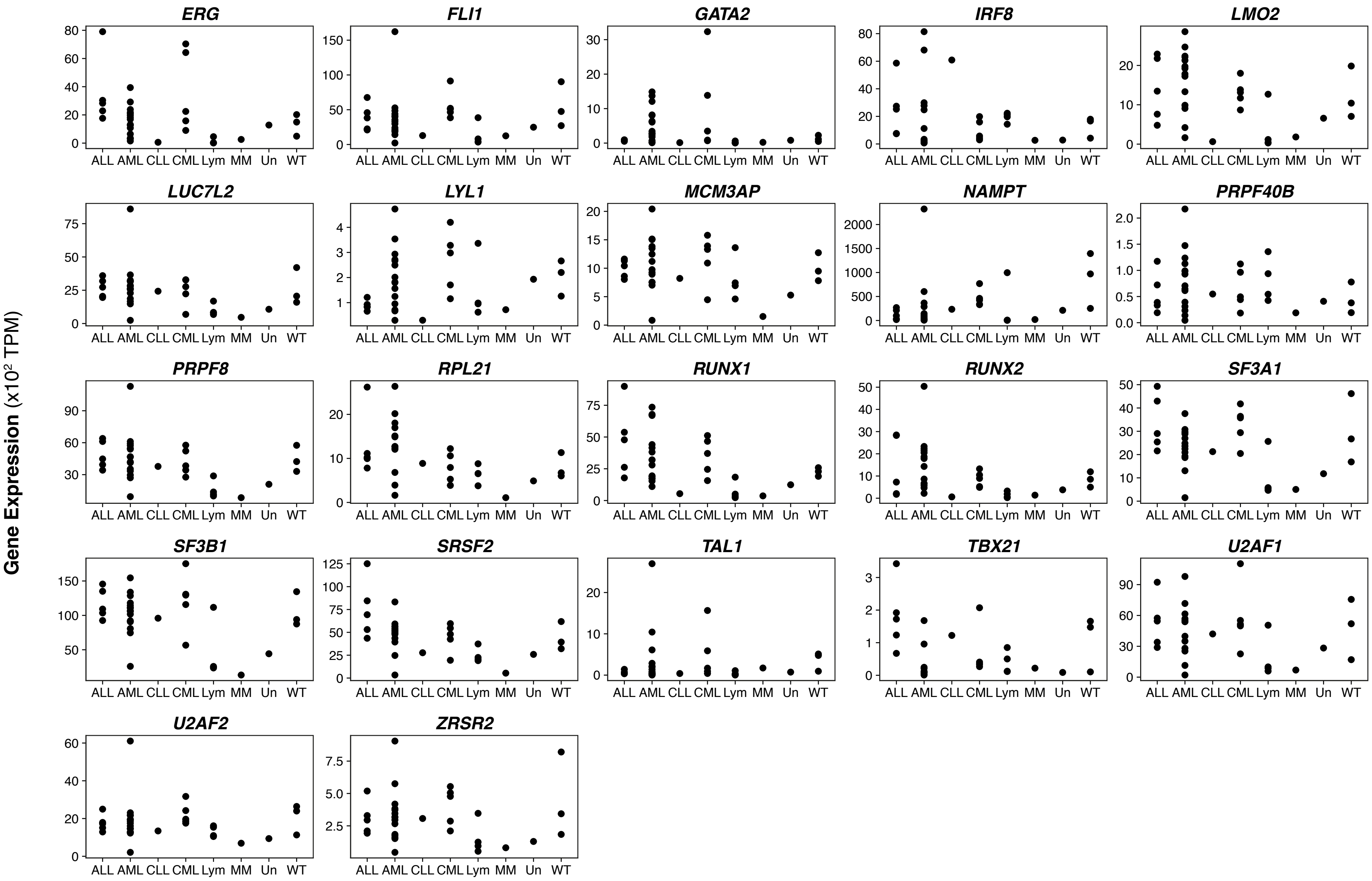

Supplementary Figure 12. Marker, transcription and splicing factor gene expression in clinical cohort samples processed on the blood panel. ALL = acute lymphoblastic leukaemia; AML = acute myeloid leukaemia; CLL = chronic lymphocytic leukaemia; CML = chronic myeloid leukaemia; Lym = lymphoma; MM = multiple myeloma; Un = uncategorised blood cancer; WT = healthy individuals.
