# Supplementary material for: Diagnosis of fusion genes using targeted RNA sequencing
Source: Nat Commun. 2019 Mar 27;10:1388. doi: 10.1038/s41467-019-09374-9 (PMC6437215; doi:10.1038/s41467-019-09374-9)
Supplement: Supplementary file 10 — Description of Additional Supplementary Files [file 41467_2019_9374_MOESM10_ESM.docx]

**Title: Supplementary Data 1.
Description:** Genes targeted by the blood panel.

**Title: Supplementary Data 2.
Description:** Genes targeted by the solid panel.

**Title: Supplementary Data 3.
Description:** Overview of patient samples and mapping statistics. BMA = bone marrow aspirate; FFPE = formalin fixed, paraffin embedded; FFT = fresh frozen tissue; PB = peripheral blood; IHC = immunohistochemistry; TKI = tyrosine kinase inhibitor. * indicates single capture instead of typical double capture; ° indicates fusion identified by either STARfusion or FusionCatcher, but not both. On-panel % indicates the percentage of uniquely mapping reads overlapping the genomic regions targeted by the capture panel. For fusion genes with multiple fusion junction isoforms, the various genomic coordinates are separated by a '|'. Samples with >1 fusion gene occupy multiple lines, one for each fusion gene.

**Title: Supplementary Data 4.
Description:** Reproducibility experiment with patient samples.

**Title: Supplementary Data 5.
Description:** V(D)J rearrangements in cell lines.

**Title: Supplementary Data 6.
Description:** Immune cell clonal abundance in patient samples. * indicates single capture instead of typical double capture.

**Title: Supplementary Data 7.
Description:** Blacklisted fusion genes removed from FusionCatcher and STARfusion default output.

**Title: Supplementary Data 8.
Description:** Overview of false positive fusion genes. For fusion genes with multiple fusion junction isoforms, the various genomic coordinates are separated by a '|'.
