# Supplementary material for: Diagnosis of fusion genes using targeted RNA sequencing
Source: Nat Commun. 2019 Mar 27;10:1388. doi: 10.1038/s41467-019-09374-9 (PMC6437215; doi:10.1038/s41467-019-09374-9)
Supplement: Supplementary file 12 — Reporting Summary [file 41467_2019_9374_MOESM12_ESM.pdf]

nature research

Corresponding author(s): Tim R. Mercer and James Blackburn

Reporting Summary

Nature Research wishes to improve the reproducibility of the work that we publish. This form provides structure for consistency and transparency in reporting. For further information on Nature Research policies, see Authors & Referees and the Editorial Policy Checklist.

Statistical parameters

When statistical analyses are reported, confirm that the following items are present in the relevant location (e.g. figure legend, table legend, main text, or Methods section).

n/a	Confirmed	
☒	☐	The exact sample size (*n*) for each experimental group/condition, given as a discrete number and unit of measurement
☒	☐	An indication of whether measurements were taken from distinct samples or whether the same sample was measured repeatedly
☒	☐	The statistical test(s) used AND whether they are one- or two-sided<br>*Only common tests should be described solely by name; describe more complex techniques in the Methods section.*
☒	☐	A description of all covariates tested
☒	☐	A description of any assumptions or corrections, such as tests of normality and adjustment for multiple comparisons
☒	☐	A full description of the statistics including central tendency (e.g. means) or other basic estimates (e.g. regression coefficient) AND variation (e.g. standard deviation) or associated estimates of uncertainty (e.g. confidence intervals)
☒	☐	For null hypothesis testing, the test statistic (e.g. *F*, *t*, *r*) with confidence intervals, effect sizes, degrees of freedom and *P* value noted<br>*Give P values as exact values whenever suitable.*
☒	☐	For Bayesian analysis, information on the choice of priors and Markov chain Monte Carlo settings
☒	☐	For hierarchical and complex designs, identification of the appropriate level for tests and full reporting of outcomes
☒	☐	Estimates of effect sizes (e.g. Cohen's *d*, Pearson's *r*), indicating how they were calculated
☒	☐	Clearly defined error bars<br>*State explicitly what error bars represent (e.g. SD, SE, CI)*

Our web collection on statistics for biologists may be useful.

Software and code

Policy information about availability of computer code

Data collection	Illumina CASAVA
Data analysis	Tally v15-065; cutadapt v1.14; STAR v2.4.2a_modified; samtools v1.5; bedtools v2.25.0; RSEM v1.3.0; FusionCatcher 0.99.6a beta; STAR-Fusion v0.3.1 Pre-Release; htseq-count 0.6.0; Stringtie v1.3.3b; IMSEQ v1.1.0-linux64; MiXCR v2.1.3; ngsplot v2.61; WebLogo 3; R v3.4.3; pheatmap 1.0.12; ggplot2 2.2.1.9000; cowplot 0.9.2

For manuscripts utilizing custom algorithms or software that are central to the research but not yet described in published literature, software must be made available to editors/reviewers upon request. We strongly encourage code deposition in a community repository (e.g. GitHub). See the Nature Research guidelines for submitting code & software for further information.

Data

Policy information about availability of data

All manuscripts must include a data availability statement. This statement should provide the following information, where applicable:

- Accession codes, unique identifiers, or web links for publicly available datasets
- A list of figures that have associated raw data
- A description of any restrictions on data availability

Sequencing data have been deposited in the NCBI Short Read Archive (SRA) with the accession code SRP156411 (BioProject PRJNA484669).

Field-specific reporting

Please select the best fit for your research. If you are not sure, read the appropriate sections before making your selection.

☒ Life sciences ☐ Behavioural & social sciences ☐ Ecological, evolutionary & environmental sciences

For a reference copy of the document with all sections, see nature.com/authors/policies/ReportingSummary-flat.pdf

Life sciences study design

All studies must disclose on these points even when the disclosure is negative.

Sample size	No sample size calculation was performed, as this proof-of-concept work was limited to the samples we were able to ethically access.
Data exclusions	No data were excluded from analyses
Replication	3 samples were chosen for replication study, prepared in triplicate and processed in triplicate for a total of 27 samples. The expected fusion genes were identified in every sample.
Randomization	Randomization is not relevant to our study, as sample allocation into groups was determined by cancer type or fusion junction location.
Blinding	During fusion gene identification, bioinformaticians were blinded to the identify of the previously-identified fusion genes and gene rearrangements.

Reporting for specific materials, systems and methods

Materials & experimental systems

n/a	Involved in the study	
☐	☒	Unique biological materials
☒	☐	Antibodies
☐	☒	Eukaryotic cell lines
☒	☐	Palaeontology
☒	☐	Animals and other organisms
☐	☒	Human research participants

Methods

n/a	Involved in the study	
☒	☐	ChIP-seq
☒	☐	Flow cytometry
☒	☐	MRI-based neuroimaging

Unique biological materials

Policy information about availability of materials

Obtaining unique materials	Targeted RNA sequencing panels available for purchase from Roche/Nimblegen

Eukaryotic cell lines

Policy information about cell lines

Cell line source(s)	ATCC
Authentication	None of the cell lines were authenticated by our facility.
Mycoplasma contamination	All cell lines tested negative for mycoplasma contamination.
Commonly misidentified lines (See ICLAC register)	none used

Human research participants

Policy information about studies involving human research participants

Population characteristics	Samples were selected based on cancer diagnosis, independent of age or gender, from previously collected research cohorts.

Recruitment

Patient samples were chosen based on cancer diagnosis and no other criteria
